# The enduring effect of education-socioeconomic differences in disability trajectories from age 85 years in the Newcastle 85+ Study

**DOI:** 10.1016/j.archger.2015.02.006

**Published:** 2015

**Authors:** Andrew Kingston, Karen Davies, Joanna Collerton, Louise Robinson, Rachel Duncan, Thomas B.L. Kirkwood, Carol Jagger

**Affiliations:** a Institute of Health and Society, Newcastle University, Baddiley-Clark Building, Richardson Road, Newcastle upon Tyne NE2 4AX, United Kingdom; b Insititue of Cellular Medicine, William Leech Building, Newcastle University, Newcastle upon Tyne NE2 4HH, United Kingdom

**Keywords:** Disability, Newcastle 85+ Study, The very old, Longitudinal studies, Education, Trajectories

## Abstract

**Objective:**

Little is known about disability progression in very old age despite this being vital for care planning. We investigate whether distinct trajectories of disability are evident from age 85 to 90 and their association with socio-economic status (SES).

**Methods:**

The Newcastle 85+ Study recruited people born in 1921 through participating general practices in Newcastle and North Tyneside. Participants underwent a health assessment (HA) at baseline, 18, 36 and 60 months and a GP record review (GPRR) at baseline, 36 and 60 months. Disability was measured via difficulty in 17 Activities of Daily Living. Trajectory identification was assessed by gender stratified, mortality adjusted, group-based trajectory modelling (GBTM) and the impact of life-course SES (level of education; occupational class; deprivation) on trajectory membership evaluated (adjusting for confounding variables).

**Results:**

851 participants agreed to HA and GPRR, 840 (98.7%) with complete disability data. Four distinct trajectories were evident for both sexes. A disability-free trajectory between age 85 and 90 was identified in men only (9% of the sample). The most disabled trajectories had severe disability at age 85 progressing to profound disability by age 90. After adjusting for confounders education remained significant; men and women with most education being less likely to be in the most disabled trajectory (Men: OR = 0.80, 95% CI 0.65–0.98; women: OR = 0.59, 95% CI 0.42–0.83).

**Conclusion:**

Distinct disability trajectories are evident in the very old and these are influenced by education, suggesting SES disadvantages cumulate throughout the life-course to create health and mortality inequalities later.

## Highlights

• Even at aged 85, four gender specific trajectories of disability are evident.
• A disability-free trajectory is found in men only comprising 9% of the male sample.
• Less education is associated with being more disabled in later life.

## 1 Introduction

Knowledge of how disability changes with advancing age is important not only for allocating the health and care resources required for our rapidly growing aging populations, but also for individuals and families to plan for increasing dependency and moves to assisted living environments. Disability in later life is affected by experiences throughout the life course, including socio-economic status as measured by education, income, or occupation (Verbrugge, Reoma, & Gruber-Baldini, 1994). SES is a strong predictor of disability onset and mortality, as well as the combined measure of disability-free life expectancy (Jagger et al., 2007; Lynch, 2008; Marmot, Shipley, Brunner, & Hemingway, 2001; Marmot & Martin, 1996; Montez, Hayward, Brown, & Hummer, 2009; Stringhini et al., 2011). More years of education are particularly associated with slower declines in disability prevalence, lower incidence and greater recovery over time (Jagger et al., 2007). Education is one factor that will change predictably as statutory school leaving ages in the United Kingdom have increased and future cohorts of older people, especially women, who have had greater access to higher education.

Mechanisms linking education and disability are ostensibly associated with behaviors that impact risk factor decision-making, mastery over one's life and/or postponed gratification (Freedman & Martin, 1999). Two popular hypotheses seek to explain the mechanisms driving the impact of SES on health in old age: the ‘cumulative disadvantage’ hypothesis and the ‘age-as-leveler’ hypothesis. The cumulative disadvantage hypothesis posits that socio-economic disparities amplify across the life course, largely as a result of differential exposure to risk factors associated with low SES, for example smoking, alcohol consumption, occupation, education and physical exercise (O’Rand, 2002). The cumulative insult of negative health behaviors/circumstances associated with low SES creates the health and mortality discrepancy. In contrast, the effect of differential SES exposures may be leveled out over the life course, perhaps due to those with low SES dying. In addition, age can bring with it many challenges in terms of sustaining homeostatic equilibrium across many body systems. This could serve to outweigh the differential impact of SES exposures which produces divergent health trajectories in younger cohorts as, in older cohorts, age-related biological forces become more influential determinants of poor health and mortality. This is known as the ‘age-as-leveler’ hypothesis (Lynch, 2008).

Most disability research focuses on onset/incidence, prevalence, or transition, and has been conducted mainly in the younger old age group (Chiu & Wray, 2011; Taylor, 2004; Verbrugge et al., 1994). There is limited research addressing disability from a pathway or trajectories perspective, particularly in the very old (aged 85 and older). The majority of previous trajectory analyses have used growth curve modeling or subjective pathway classification, both of which have limitations (Ferrucci et al., 1996; Taylor & Lynch, 2004; Zimmer, Martin, Nagin, & Jones, 2012). Furthermore, many studies fail to fully account for loss to follow up (through death or withdrawal, both of which occur more often in the very old) with a resulting bias (Wolinsky, Armbrecht, & Wyrwich, 2000). In this paper we explore associations between SES and disability trajectories, specifically in the very old, using data from the Newcastle 85+ Study; we use group-based trajectory modelling to improve upon previous analyses (Nagin, 2005). The common underlying assumptions of the majority of previous analyses center on the distribution of trajectory parameters and require these to follow a continuous multivariate normal distribution. The technique we use (GBTM) is less restrictive and allows for clusters of unique developmental trajectories that are potentially a function of different disability aetiologies, thus giving scope to further understand the disability process in the very oldOur paper has two objectives. Firstly, we investigate for the first time whether distinct trajectories of disability are evident in a cohort of the very old, after accounting for mortality. Secondly, we examine the extent to which early, mid and/or late life SES predicts specific disability trajectories. We hypothesise that if the age-as-leveler theory is true, then early-life markers of SES will not prove differential across trajectories in the very old. Conversely, if the cumulative disadvantage hypothesis is true then SES throughout the life course will associate with disability patterns in the very old.

## 2 Methods

### 2.1 Participants

Data were drawn from the Newcastle 85+ Study; full details of the study design, protocol and participant recruitment have been described previously (Collerton et al., 2009). In brief, this is a longitudinal study of adults in Newcastle upon Tyne and North Tyneside (North-East England) who was born in 1921, who turned 85 years of age in 2006 when recruitment commenced, and who were registered with a participating general practice. At baseline (wave 1), trained research nurses carried out a detailed multi-dimensional health assessment (MDHA) of participants in their own home or other permanent place of residence (including institutional care settings) together with a detailed review of their general practice medical records (GPRR). Follow up MDHAs were carried out at 18, 36 and 60 months post-baseline with a further GPRR at 36 and 60 months.

### 2.2 Disability

Disability was assessed at baseline and all follow-up MDHAs through participants’ self-report of their ability to perform 17 Instrumental and Basic Activities of Daily living (IADLs and BADLs) (Katz, Ford, Moskowitz, Jackson, & Jaffe, 1963; Lawton & Brody, 1969) (Supplementary Fig. 1). Participants scored one for each activity they had any difficulty with and zero for each activity performed without difficulty; summation over the 17 activities produced a total disability score (range 0–17) with a higher score indicating a higher level of disability. Activities which predominantly involved mobility (getting around the house, getting in and out of a chair, shopping, going up and down stairs, walking at least 400 yards) were highly correlated with objectively measured timed-up-and-go test times for both men and women (Jagger et al., 2011).

### 2.3 Mortality

Date and cause of death were obtained through the Health and Social Care Information Service Centre. Survival time was constructed from date of baseline MDHA to date of death and censored at wave 4 (60 months from baseline). For the purposes of this analysis we considered all-cause mortality.

### 2.4 Measures of socio-economic status

Early-life SES was measured through the number of years of full-time education. Mid-life SES was assessed by main working life occupation, classifying participants through the National Statistics Socio-economic Classification system (NS-SEC) into three categories (routine and manual occupations, intermediate occupations and higher managerial, administrative and professional occupations) (ONS, 2010). As a proxy for current (late-life) SES we derived the area Index of Multiple Deprivation (IMD) from participants’ postcodes; this combines a number of indicators chosen to reflect a range of economic, social and housing issues into a single deprivation score with higher scores representing those living in more deprived areas (and therefore greater disadvantage) (Office of the Deputy Prime Minister, 2004).

### 2.5 Confounding variables

Models were adjusted for some of the major factors associated with both disability and SES: disease burden, Body Mass Index (BMI); and depressive symptomatology. Presence of specific diseases during the participants’ lifetime was recorded in the GPRR and disease burden calculated as the number of diseases present from a list of the eight most prevalent (Kingston et al., 2014) (Supplementary Fig. 2). BMI was calculated from height (derived from demi-span) and weight. Depressive symptomatology was measured using the 15 item Geriatric Depression Scale (Yesavage & Brink, 1983).

### 2.6 Statistical methods

Gender differences in SES and key health characteristics were assessed as follows: education, IMD (ordinal logistic regression); NS-SEC (multinomial logistic regression); disease count, BMI (*t*-test); and disability (Tobit regression to account for the floor effects (Austin, Escobar, & Kopec, 2000)). To explore patterns of individual trajectories of disability we used group-based trajectory modelling (Nagin, 2005). This technique first determines the number of distinct trajectories via polynomial functions in time using a censored normal distribution. Non-random subject attrition, in particular due to mortality, was accounted for by a group-specific function linked to the probability of death by age (Haviland, Jones, & Nagin, 2011). We explored a number of trajectory models with the best fitting model selected by the Bayesian Information Criterion (BIC) and the fit further assessed by ensuring the posterior probability of group membership for all participants exceeded 70%. All participants satisfied this condition in the final model (Nagin, 2005).

We examined the effect of SES measures on the disability trajectories by multinomial logistic regression, first fitting SES measures singly, then with adjustment for confounders, and finally with all SES measures together. As we have previously shown in this cohort that females are at a disadvantage in terms of disability (Collerton et al., 2009), we fitted trajectory models separately for men and women. Sensitivity analyses were undertaken to determine the effects of combining both mortality and participants lost to follow-up into one category. Analyses were carried out in Stata 12.1 (StataCorp. 2011. *Stata Statistical Software: Release 12*. College Station, TX: StataCorp LP.) and the SAS^®^ Trajectory Procedure (Jones, Nagin, & Roeder, 2001) on the SAS^®^ platform (v9.2).

### 2.7 Role of the funding source

The funders had no role in study design, data collection, data analysis, data interpretation, or writing of the report. The corresponding author had full access to all the data and responsibility for the decision to submit for publication.

## 3 Results

A total of 851 Newcastle 85+ Study participants underwent both the MDHA and GPRR at baseline (wave 1); of these 840 had complete disability data, with 63.1% (*n* = 540) being female. Disability level increased from age 85 (wave 1) to 90 years (wave 4) for both men and women and was consistently and statistically significantly greater at each wave for women compared to men (Table 1). Compared to men, women were more likely to reside in a more deprived area (OR: 1.35, 95% CI: 1.04–1.76) and to have worked in intermediate occupations (OR: 2.58, 95% CI: 1.57–4.26). No gender differences were detected in the levels of education or disease count at baseline (Table 1). The retention profile of participants across the course of the study (to wave 4) is included in Table 2.

### 3.1 Disability trajectories

**Fig. 1:**
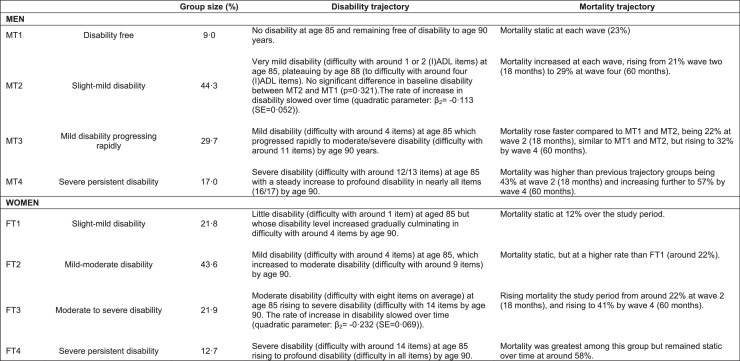
Descriptions of disability trajectories for men and women.

**Fig. 2:**
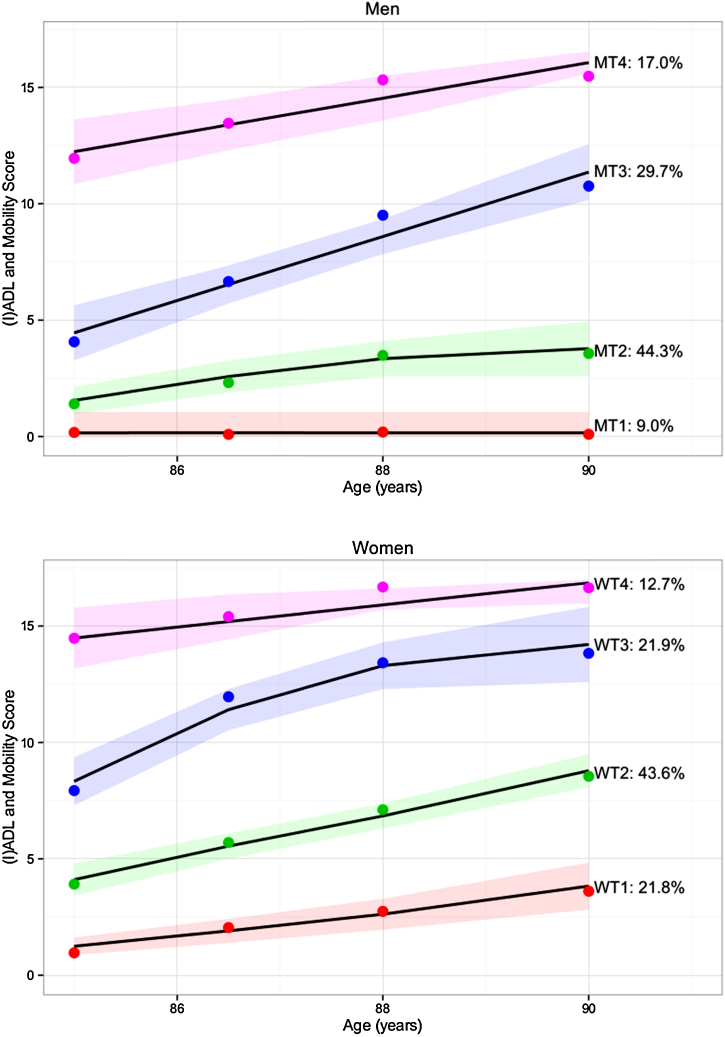
Disability trajectories*.

For both sexes, disability trajectories were best represented by a four-group model (model parameters shown in Supplementary Table 1). In women, the four trajectories (WT1–WT4) showed a gradual increase in the level of disability with advancing age and 21.8%, 43.6%, 21.9% and 12.7% were classified from WT1-WT4 respectively. Three trajectories were ascertained for men, showing monotonically increasing disability, with 44.3%, 29.7% and 17.0% being classified from MT2-MT4. However, trajectory one (MT1), comprising 9% if the male sample remained free of disability to at least aged 90 years. For both men and women, trajectory two contained the most participants. For men this was those people who experienced slight to mild disability (44.3%) and for women it was those who experienced mild to moderate disability (43.6%). A group with severe, persistent disability (initially dependent in 12 or more (I)ADLs) was evident for both sexes, though their mortality experience differed by sex with increasing mortality over time in men and static mortality in women. The four trajectories for men and women are described in detail in Fig. 1 and illustrated graphically in Fig. 2 (upper panel: men, lower panel: women). Supplementary Fig. 3 details the mortality trajectories by gender.

Combining mortality and those who withdrew to investigate the impact from two sources of attrition did not alter the number or shape of the trajectories.

### 3.2 The impact of SES on disability trajectories

We first examined the impact of the three SES measures individually (Table 3 model 1). Men and women with more education were less likely to belong to the more disabled trajectories with a stronger education gradient in women than men. Those with 12 or more years of education were less likely to belong to the most disabled trajectory compared to the least (Men: OR = 0.69, 95% CI 0.51–0.93; women: OR = 0.54, 95% CI 0.30–0.96) and women with the least education (0–9 years) were more likely to be in the most disabled (FT4) than the least disabled (FT1) trajectory (OR = 1.21, 95% CI 1.01–1.45). With regard to mid-life SES, identical patterns prevailed. Men and women who had been in managerial occupations were less likely to belong to the most disabled trajectory (Men: OR = 0.33, 95% CI 0.15–0.71; women: OR = 0.33, 95% CI 0.21–0.51) and women from manual occupations were more likely to be in the most disabled (FT4) than the least disabled (FT1) trajectory (OR = 1.35, 95% CI 1.05–1.74). Late-life socio-economic status (IMD) only impacted men, with those in the least deprived quartile of IMD being less likely to be in the most disabled trajectory (MT4) compared to the least disabled (MT1) (OR = 0.42, 95% CI 0.31–0.57).

When all socio-economic status indicators (i.e. early, mid and late life) were included in the model (model 2) only the effect of education remained significant and this effect persisted, though attenuated, after adjustment for potential confounders (disease burden, BMI, depressive symptomatology). Thus men and women with the most education remained less likely to be in the greatest, compared to the least, disabled trajectory (Men: OR = 0.80, 95% CI 0.65–0.98; women: OR = 0.59, 95% CI 0.42–0.83).

## 4 Discussion

We used group-based trajectory modelling to investigate whether distinct disability trajectories were present for very old men and women, and the effect of life course SES. Four distinct disability trajectories were evident for men and women, differentiated both by the initial level of disability and the pace of progression. Only in men did we detect a group (comprising 9%) who remained free of disability from age 85 to 90. Moreover, although the effects of SES in mid-life (occupationally based) and late-life (area deprivation) on trajectory membership were attenuated after adjustment for potential confounders, the effect of early-life SES (education) remained, with men and women with the more education (12+ years) being significantly less likely to be in the most disabled trajectories.

Despite the disability free trajectory being absent in women, there were similarities in the initial level and progression of the remaining trajectories between the sexes. Trajectories MT2 and MT3 for men (slight to mild disability and mild progressing to moderate respectively) were equivalent to the first two trajectories in women (FT1 and FT2), whilst the last trajectory for men (MT4: severe persistent disability) was between the final two trajectories for women (FT3, FT4), these being differentiated by initial disability level (moderate versus severe) but all showing the effect of reaching a plateau in disability level by age 90.

Our analytic technique, group-based trajectory modelling, accounted for non-random subject attrition (mortality) and this reaffirmed that mortality and disability are intricately linked. As the level of disability increased within a trajectory, mortality also increased with its functional form aligned with that of the disability trajectory, i.e. mortality was a function of disability severity. Although male mortality is known to exceed that of women of the same age, the probability of death occurring before participation in the next wave was almost identical for men and women in similar trajectories. For example men in MT3 and women in FT2 both had a 22% chance of dying prior to wave 2 (age 86.5 years). It may be possible that, as the more acutely fatal conditions become less common, and men suffer long-term disabling conditions, that their mortality experience begins to resemble that of women. This would explain, at least in part, the more rapid increase in male compared to female life expectancy and the subsequent narrowing of the gender gap.

There are two main limitations to our study. Firstly, although the time interval between disability measures was only 18 months for the first three study waves, we may have missed some disability transitions which could have resulted in fluctuating trajectories. However such fluctuations may be noise around an otherwise steady downward progression. Secondly, our study relied on proxy measures of disadvantage earlier in life (education and occupation) in contrast to cohort studies which follow individuals from birth and which can collect contemporaneous data to measure disadvantage. Education and occupation are unlikely to be subject to recall bias but they cannot capture the whole picture of early-life disadvantage.

Strengths of our study include: the large number of individual ADL items constituting the disability score, thus providing a greater spectrum of disability; validation of the self-report ADL items with the objectively measured TUG; comprehensive follow-up of the study participants with little attrition other than death; and the study design of a single birth cohort of a total population (community dwelling: inclusive of those living in care homes (nursing/residential) who are socio-demographically nationally representative (Collerton et al., 2009; Jagger et al., 2011).

There is little research examining disability trajectories, and even less that focuses on the very old including those living in institutional care. Using similar techniques to ours to account for decedents but with fewer measures of ADL limitations, a study of the very old in China also identified a group of consistently non-disabled men between the ages of 80 and 90 (Zimmer et al., 2012), lending credence that this able group of men may exist in other populations regardless of geographical location. On the other hand older people surviving with persistent severe disability, as ours, have been identified in the US, although this study was restricted to community-dwelling older people aged 70 or more years interviewed monthly, not accounting for mortality (Gill, Gahbauer, Han, & Allore, 2010). Disability has been found to be a dynamic process over short periods of time and we have shown that this dynamism relaxes long term to form distinct trajectories. The number of trajectories we found is broadly consistent with other literature in younger ages and they are developmentally similar (Gill, Gahbauer, Lin, Han, & Allore, 2013; Han et al., 2013; Hardy, Dubin, Holford, & Gill, 2005). Our analyses revealed a disability-free trajectory in men but not women, and a persistently-disabled trajectory in women but not men; these gender differences suggest that analysis of men and women together might mask gender specific trajectories.

The impact of SES on future health and functional status is widely researched in the younger old but there is a dearth of information in the very old. We have shown that early-life SES (education) still determines disability trajectories after age 85. Though mid (occupation) late-life SES (deprivation) gave similar pictures when assessed individually, only education remained significant when all SES variables were included and confounders adjusted for. Consistent with other research, having more education was significantly associated with less disabled trajectories at aged 85, irrespective of gender (Freedman & Martin, 1999; Hayward & Gorman, 2004; Taylor, 2010). Our results lend credibility to the cumulative disadvantage hypothesis whereby those disadvantaged by less education in early life are potentially exposed to a greater degree of social inequality thereafter, and suggest that this inequality reaches right through the life course, influencing disability pathways beyond age 85. Conversely, we found no evidence that biological forces move to neutralise the impact of SES disparities in the very old (i.e. the age as leveler theory) and that disability in very late life is not simply explained by a person's disease profile. This suggests that future cohorts of very old people may be less disabled since they will have enjoyed more years of education.

While it is an interesting finding that we discovered 9% of men remained disability free over the course of the study, it is important to note that all other participants showed increasing levels of disability over time. If these trajectories remain static over the course of the next fifteen years (to 2030) and with the increases in the very old population (aged 85 and over: 47.3% for men and 38.6% for women) we will see increases in the region of 50,000 people (in the UK) who belong to the most disabled trajectories (WT3/MT3–WT3/MT4). This will have important implications for policy makers and health care providers to ensure services have the capacity to cope with this increase. However, further exploration of the trajectories in terms of their disease, syndromes and psychosocial profile could yield important information (in terms of etiology and identification of risk factors) and increase our understanding of the disability process, leading to potential interventions that could positively augment the disability trajectories themselves.

## 5 Conclusion

In summary, four trajectories of disability are able to describe both men and women in our cohort of very old people. Of these trajectories, we detected a disability-free trajectory only in men. We found that early life SES (education) was associated with trajectory affiliation at age 85, with those less educated more likely to be in the most disabled trajectory, even after adjusting for multiple confounding variables. Our findings add strength to the theory that SES accumulates over the life course (cumulative disadvantage theory) and that disability at later ages is not simply a result of age related biological decline. Furthermore, it suggests that future cohorts of the very old with more education could enjoy less severe disability trajectories as they age.

## Conflict of interest statement

All authors declare no conflict of interest.

## Figures and Tables

**Table 1:** Socio-economic and key health characteristics of study sample.

	Men	Women	All	Gender difference *p*-value	Magnitude of gender difference
Gender – % (*n*)	38.10 (320)	61.90 (520)	100.00 (840)	–	–
Education (no. of years) – % (*n*)					
0–9	61.78 (194)	65.69 (335)	64.20 (529)	0.3040	0.86 (0.65–1.16)^a^
10–11	24.84 (78)	21.57 (110)	22.82 (188)		
12+	13.38 (42)	12.75 (65)	12.99 (107)		
NS-SEC 3 – % (*n*)					
Routine occupations	53.05 (165)	51.03 (247)	51.82 (412)	–	Referent^b^
Intermediate occupations	7.400 (23)	18.39 (89)	14.09 (112)	<0.001	2.58 (1.57–4.26)
Professional/managerial occupations	39.55 (123)	30.58 (148)	34.09 (271)	0.1670	0.80 (0.59–1.10)
Deprivation (IMD) – % (*n*)					
>75th centile	22.81 (73)	25.58 (133)	24.52 (206)	0.0250	1.35 (1.04–1.76)^a^
25th ≤ centile ≤ 75th	46.88 (150)	52.50 (273)	50.36 (423)		
<25th centile	30.31 (97)	21.92 (114)	25.12 (211)		
Disability score – median (IQR)					
Wave 1 (*n* = 840)	2 (0–6)	4 (1–8)	3 (1–8)	<0.001	2.23 (1.39–3.07)^c^
Wave 2 (*n* = 625)	4 (1–8)	6 (3–10)	5 (2–9)	<0.001	2.13 (1.24–3.02)^c^
Wave 3 (*n* = 480)	5 (2–10)	7 (4–11)	6 (3–10)	0.0010	1.49 (0.48–2.51)^c^
Wave 4 (*n* = 341)	5 (2–9)	7 (4–11)	7 (3–11)	0.0010	2.03 (0.76–3.29)^c^
Depression (15 item GDS) – median (IQR)	3 (1–4)	3 (2–5)	3 (2–5)	0.0057	–
Disease count – mean (SD)	2.15 (1.35)	2.08 (1.33)	2.11 (1.34)	0.5066	0.06 (−0.12 – 0.25)^d^
Body mass index – mean (SD)	24.58 (3.82)	24.42 (4.73)	24.49 (4.39)	0.6286	0.16 (−0.48–0.81)^d^

a Ordinal logistic regression.

b Multinomial logistic regression.

c Tobit regression.

d *T*-test.

**Table 2:** Participant retention profile.

	Participant	Died	Withdrawn
Baseline			
Men	38.10 (320)	–	–
Women	61.90 (520)	–	–
Wave 2			
Men	73.13 (234)	19.69 (63)	7.19 (23)
Women	75.38 (391)	13.46 (70)	11.35 (59)
Wave 3			
Men	55.00 (176)	32.50 (104)	12.50 (40)
Women	58.46 (304)	23.65 (123)	17.88 (93)
Wave 4			
Men	36.88 (118)	48.75 (156)	14.38 (46)
Women	42.88 (223)	35.19 (183)	21.92 (114)

**Table 3:** Multinomial logistic regression of the impact of SES variables on the trajectory of disability by gender–Odds Ratio (95% CI).

	Men			Women		
	MT2 vs. MT1	MT3 vs. MT1	MT4 vs. MT1	MT2 vs. MT1	MT3 vs. MT1	MT4 vs. MT1
Model 1^b^						
Education (no. of years)						
0–9	1.02 (0.82–1.27)	1.21 (0.32–4.58)	1.23 (0.26–5.82)	0.98 (0.38–2.53)	1.01 (0.52–1.96)	1.21 (1.01–1.45)^a^
10–11	Referent			Referent		
12+	0.92 (0.54–1.57)	0.99 (0.21–4.67)	0.69 (0.51–0.93)^a^	0.93 (0.31–2.79)	0.73 (0.54–0.98)^a^	0.54 (0.30–0.96)^a^
Occupational class						
Routine and manual	0.88 (0.39–1.99)	1.03 (0.41–2.59)	1.01 (0.38–2.68)	1.00 (0.45–2.22)	1.02 (0.68–1.53)	1.35 (1.05–1.74)^a^
Intermediate	Referent			Referent		
Managerial	1.21 (0.45–3.25)	0.84 (0.21–3.36)	0.33 (0.15–0.71)^a^	0.96 (0.21–4.39)	0.82 (0.34–1.98)	0.33 (0.21–0.51)^a^
Deprivation (IMD)						
> 75th centile	0.84 (0.21–3.36)	0.98 (0.51–1.88)	1.19 (0.87–1.63)	1.05 (0.81–1.36)	1.06 (0.72–1.56)	1.15 (0.81–1.63)
25th ≤ centile ≤ 75th	Referent			Referent		
<25th centile	0.87 (0.11–6.88)	0.99 (0.51–1.92)	0.42 (0.31–0.57)^a^	1.02 (0.79–1.32)	0.84 (0.51–1.38)	0.82 (0.56–1.20)

Model 2^c^						
Education (no. of years)						
0–9	0.99 (0.51–1.92)	0.99 (0.48–2.04)	1.09 (0.84–1.41)	1.04 (0.41–2.64)	1.03 (0.46–2.31)	1.12 (0.81–1.55)
10–11	Referent			Referent		
12+	0.92 (0.73–1.16)	0.88 (0.62–1.25)	0.71 (0.55–0.92)^a^	1.02 (0.51–2.04)	0.62 (0.32–1.20)	0.55 (0.41–0.74)^a^
Occupational class						
Routine and manual	0.98 (0.42–2.29)	1.12 (0.64–1.96)	1.14 (0.79–1.65)	1.05 (0.59–1.87)	1.03 (0.51–2.08)	1.11 (0.63–1.96)
Intermediate	Referent			Referent		
Managerial	0.91 (0.31–2.67)	0.90 (0.42–1.93)	0.82 (0.29–2.32)	0.85 (0.21–3.44)	0.89 (0.45–1.76)	0.74 (0.39–1.40)
Deprivation (IMD)						
> 75th centile	1.05 (0.52–2.12)	1.06 (0.68–1.65)	1.06 (0.78–1.44)	1.01 (0.42–2.43)	1.05 (0.11–10.02)	1.06 (0.41–2.74)
25th ≤ centile ≤ 75th	Referent			Referent		
<25th centile	1.03 (0.42–2.53)	0.92 (0.52–1.63)	0.86 (0.62–1.19)	1.11 (0.35–3.49)	0.97 (0.59–1.59)	0.91 (0.61–1.36)

Model 3^d^						
Education (no. of years)						
0–9	1.00 (0.53–1.89)	0.99 (0.69–1.42)	1.12 (0.57–2.20)	0.97 (0.35–2.69)	1.01 (0.42–2.43)	1.09 (0.63–1.89)
10–11	Referent			Referent		
12+	0.87 (0.21–3.60)	0.82 (0.54–1.25)	0.80 (0.65–0.98)^a^	0.93 (0.49–1.77)	0.86 (0.54–1.37)	0.59 (0.42–0.83)^a^
Occupational class						
Routine and manual	1.15 (0.32–4.13)	1.08 (0.41–2.84)	1.18 (0.39–3.57)	1.06 (0.88–1.28)	1.10 (0.72–1.68)	1.09 (0.76–1.56)
Intermediate	Referent			Referent		
Managerial	1.09 (0.49–2.42)	0.93 (0.42–2.06)	0.87 (0.56–1.35)	0.90 (0.51–1.59)	0.87 (0.67–1.13)	0.86 (0.64–1.16)
Deprivation (IMD)						
>75th centile	1.03 (0.47–2.26)	1.02 (0.52–2.00)	1.05 (0.43–2.56)	1.00 (0.21–4.76)	1.02 (0.35–2.97)	1.15 (0.87–1.52)
25th ≤ centile ≤ 75th	Referent			Referent		
<25th centile	0.98 (0.35–2.74)	0.98 (0.46–2.09)	0.93 (0.67–1.29)	0.93 (0.19–4.55)	0.93 (0.16–5.41)	0.89 (0.28–2.83)

a Statistically significant.

b Model 1 – each SES covariate considered alone with no-adjustment.

c Model 2 – each covariate adjusted for other SES covariates.

d Model 3 – each covariate adjusted for other SES covariates plus BMI, disease burden and depressive symptomatology.
