# Supplementary material for: The enduring effect of education-socioeconomic differences in disability trajectories from age 85 years in the Newcastle 85+ Study
Source: Arch Gerontol Geriatr. 2015 May-Jun;60(3):405–11. doi: 10.1016/j.archger.2015.02.006 (PMC4407633; doi:10.1016/j.archger.2015.02.006)
Supplement: Supplementary file 1 [file mmc1.docx]

Supplementary table 1: Disability trajectory and mortality trajectory parameters by gender

|  | **TRAJECTORY 1** | **TRAJECTORY 2** | **TRAJECTORY 3** | **TRAJECTORY 4** |
| --- | --- | --- | --- | --- |
| **MEN** | **Disability free**  **(MT1)** | **Slight-mild disability (MT2)** | **Mild disability progressing rapidly**  **(MT3)** | **Severe persistent disability (MT4)** |
| **Parameters for disability trajectory** |  |  |  |  |
| Intercept | -3·620 (1·307) | 3·121 (0·290) | 8·565 (0·347) | 15·772 (0·526) |
| Linear age (centred) |  | 0·487 (0·115) | 1·390 (0·131) | 1·105 (0·215) |
| Quadratic age (centred) |  | -0·113 (0·052) |  |  |
| **Parameters for mortality trajectory** |  |  |  |  |
| Intercept | -1·485 (0·460) | -1·652 (0·300) | -1·674 (0·760) | -2·011 (1·037) |
| Linear centred age at previous wave | 1·610 (1·049) | 0·209 (0·080) | 0·095 (0·079) | 0·143 (0·073) |
| **BIC** | -2532·22 |  |  |  |
| **WOMEN** | **Slight-mild disability**  **(FT1)** | **Mild-moderate disability (FT2)** | **Moderate to severe disability**  **(FT3)** | **Severe persistent disability (FT4)** |
| **Parameters for disability trajectory** |  |  |  |  |
| Intercept | 2·234 (0·265) | 6·901 (0·247) | 13·420 (0·377) | 16·26 (0·837) |
| Linear age (centred) | 0·699 (0·100) | 0·957 (0·077) | 1·031 (0·165) | 1·115 (0·271) |
| Quadratic age (centred) |  |  | -0·232 (0·069) |  |
| **Parameters for mortality trajectory** |  |  |  |  |
| Intercept | -2·155 (0·365) | -1·470 (0·326) | -2·797 (0·837) | 1·789 (2·012) |
| Linear centred age at previous wave | 0·087 (0·153) | 0·033 (0·052) | 0·179 (0·064) | -0·093 (0·128) |
| **BIC** | -4427·65 |  |  |  |

**Supplementary Figure 1: 17 Instrumental and Basic Activities of Daily Living (IADLs, BADLs) and mobility activities included in disability score, with possible responses**

| **Instrumental and Basic Activities of Daily Living and Mobility items** |
| --- |
| Are you able to get in and out of bed? |
| Are you able to get and out of a chair? |
| Are you able to get on and off the toilet? |
| Are you able to up and down stairs/steps? |
| Are you able to dress and undress yourself? |
| Are you able to wash your face and hands? |
| Are you able to wash yourself all over? |
| Are you able to cut your own toenails? |
| Are you able to feed yourself? |
| Are you able to cook a hot meal? |
| Are you able to shop for your groceries? |
| Are you able to do light housework? |
| Are you able to do heavy housework?  Are you able to manage your medications?  Are you able to manage money? |
| Possible responses (score): |
| *I have no difficulty doing this by myself (0)* |
| *I have some difficulty doing this by myself (1)* |
| *I can do this by myself if I use an aid or appliance (1)* |
| *I am unable to do this by myself; I need someone else's help (1)·* |

**Supplementary Figure 2: Conditions examined with data sources and ascertainment criteria**

| **Disease Group** | **Data source and ascertainment criteria** |
| --- | --- |
| **Arthritis^*^** | GP records: any recorded diagnosis of: Generalised Osteoarthritis, Hand, Hip and Knee Osteoarthritis, Rheumatoid arthritis, Degenerative arthritis, Polyarthritis, Gouty arthritis, Septic arthritis, Peri-arthritis, Lumbar Spondylosis, Cervical Spondylosis, Ankylosing Spondylitis and Psoriatic arthritis |
| **Hypertension^*^** | GP records: any recorded diagnosis of Hypertension |
| **Cardiac disease^*^** | GP records: : any recorded diagnosis of Heart Failure, Ischaemic heart disease (Angina, Myocardial Infarction, Coronary Artery Bypass Graft, Coronary Angioplasty/Stent) |
| **Respiratory disease^*^** | GP records: : any recorded diagnosis of Bronchiectasis, Pulmonary Fibrosis, Fibrosing alveolitis, Asbestosis, Pneumoconiosis, Asthma, Chronic Bronchitis, Emphysema, COPD |
| **Cerebrovascular disease^*^** | GP records: : any recorded diagnosis of Stroke, Transient Ischaemic Attack, Carotid Endarterectomy |
| **Diabetes mellitus^*^** | GP records: : any recorded diagnosis of Type I, Type II and type unspecified diabetes mellitus |
| **Cancer^*^** | GP records: any cancer diagnosis in past 5 years excluding non-melanoma skin cancer |
| **Cognitive Impairment**^†^ | Standardised Mini-Mental State Examination (sMMSE) score of ≤21 |

* Data taken from GP record review

^†^ Score calculated from multi-dimensional health assessment (sMMSE)

**Supplementary figure 3: Predicted probability of dying prior to next wave**

**
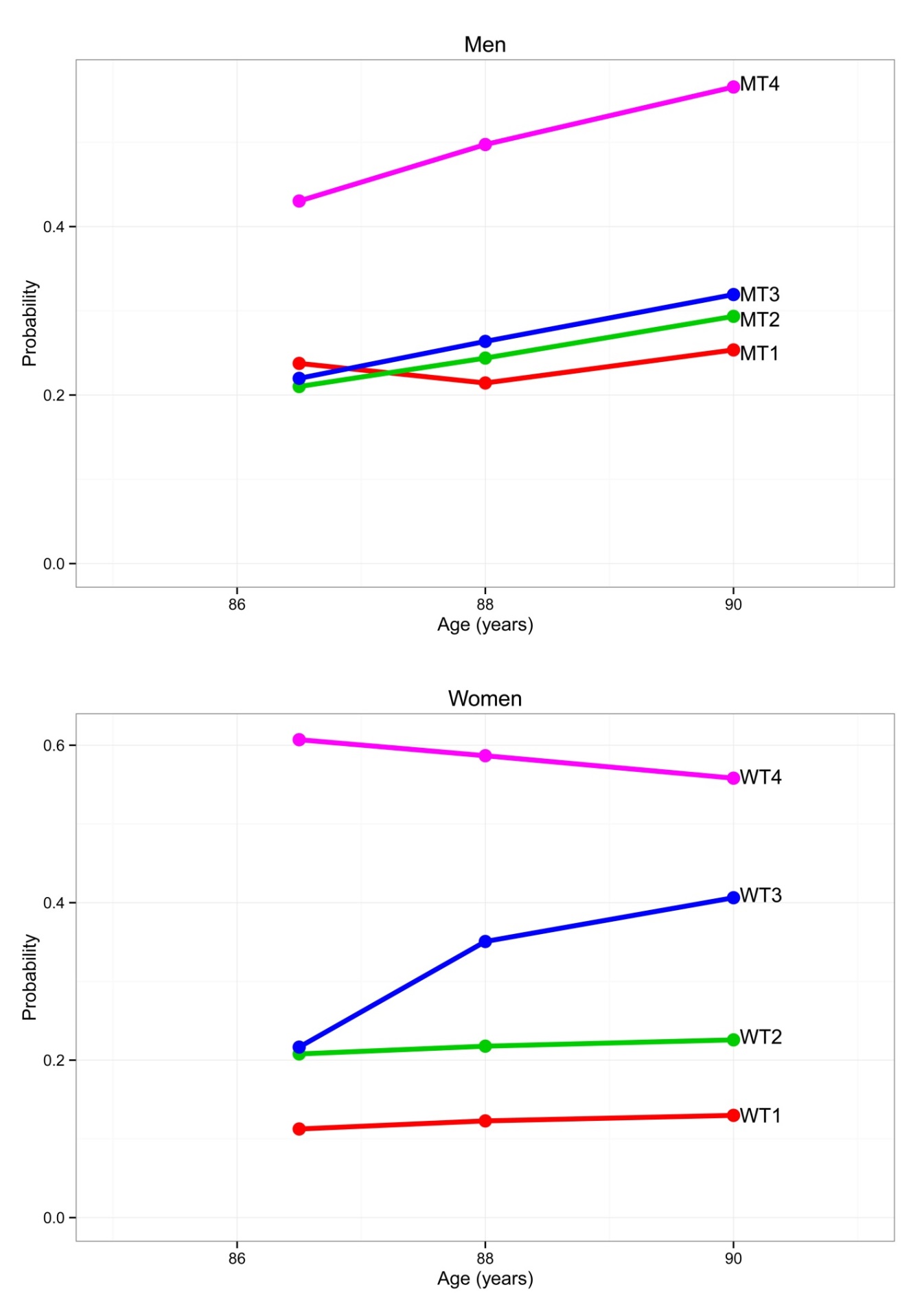
**
